# The Surgical Diseases of the Genitourinary Organs

**Published:** 1903-07

**Authors:** 


The Surgical Diseases of the Genitourinary Organs. By E. L. Keyes, A. M., M. D., LL. D., Consulting Surgeon to the Bellevue, and the Skin and Cancer Hospitals; Surgeon to St. Elizabeth Hospital, New York. And E. L. Keyes, Jr., A. B., M. D. Ph. D., Lecturer on Genitourinary Surgery, New York Polyclinic Medical School and Hospital; Surgeon to the Out-Patient Department, St. Vincent's Hospital. A revision of Van Buren and Keyes's Textbook. Octavo, pp. 843. With 174 illustrations in the text and 10 plates, 8 of which are colored. New York and London: D. Appleton & Co. 1903. [Price, $5.00.]

This treatise is a valuable addition to the works on this subject. It has been the evident aim of the authors to elucidate fully the clinico-surgical aspects of their subject. Full details are given for the use and management of every instrument and procedure necessary for the practice of genitourinary surgery. In the elimination of syphilis and the sexual psychoses from their work the authors have succeeded in presenting a model textbook on genitourinary diseases without treading on the realms of the venereal specialist.

The views of the authors are clear, concise, conservative and logical. Every side of this branch of surgery is carefully and intelligently dealt with, and each chapter contains opinions and suggestions of such a practical nature that they are sure to be of benefit to the reader. The illustrations and text are excellent, the general arrangement of the subject matter being in logical order. H.R.G.

